# ﻿Additions to Acrocalymmaceae and Didymosphaeriaceae (Pleosporales, Dothideomycetes): Some interesting novel additions from plant litter in China

**DOI:** 10.3897/mycokeys.122.163383

**Published:** 2025-09-05

**Authors:** Danushka S. Tennakoon, Nimali I. de Silva, Sinang Hongsanan, Ning Xie

**Affiliations:** 1 Shenzhen Key Laboratory of Microbial Genetic Engineering, College of Life Science and Oceanography, Shenzhen University, Shenzhen 518060, China Shenzhen University Shenzhen China

**Keywords:** Multi-gene phylogeny, new host records, new species, saprobes, systematics, taxonomy

## Abstract

During a survey of fungal diversity of plant litter in China, numerous isolates with affinity to Pleosporales (Dothideomycetes, Ascomycota) were recovered. Morphology, coupled with phylogenetic analyses using Maximum Likelihood and Bayesian Inference on combined LSU, SSU, ITS, *tub*2, and *tef*1-α sequence data, revealed that these isolates belong to the families Acrocalymmaceae and Didymosphaeriaceae, and are introduced as *Acrocalymma poaceicola***sp. nov.** and *Neptunomyces chinensis***sp. nov.** In addition, five new host records, *A. ampeli*, *Neokalmusia arundinis*, *Paraconiothyrium archidendri*, *Pseudopithomyces chartarum*, and *Spegazzinia deightonii* are reported from litter of *Livistona chinensis*, *Sporobolus alterniflorus*, *Citrus maxima*, *Hedychium coronarium*, and *Arundo pliniana*, respectively. All the new species and host records were compared with phylogenetically and morphologically closely related species, and detailed descriptions, micrographs, and phylogenetic analysis results are provided.

## ﻿Introduction

Plant litter, composed of dead plant materials, such as leaves, twigs, bark, seeds, flowers, roots, and other wood debris accumulates naturally on the forest floor (Wardle et al. 2002; Berg and McClaugherty 2008; Liu et al. 2022). Plant litter plays a fundamental role in ecosystem dynamics through the decomposition process (Schroeter et al. 2022), and is crucial for nutrient cycling, improving soil structure, enhancing biodiversity, and boosting forest productivity and resilience (Sayer 2006; Pei et al. 2017; Giweta 2020; Pascoal et al. 2021). The significance of plant litter extends beyond its ecological functions, as it also serves as a habitat for a wide range of organisms, including bacteria, fungi, and macroinvertebrates. Of them, fungal communities are regarded as the “key players” in the decomposition of plant litter, because of their capacity to produce a vast array of extracellular enzymes (Geethanjali and Jayashankar 2016; Asplund et al. 2018; Zhou et al. 2023). They are responsible for the breakdown of plant litter in various ecosystems, significantly contributing to nutrient cycling and the decomposition of organic matter (Griffiths et al. 2021; Pascoal et al. 2021; Tennakoon et al. 2021; Liu et al. 2022). Hence, it is vital to explore the fungal diversity residing in plant litter to comprehend the relationships involved in plant litter decomposition. Many researchers have conducted studies on fungal species inhabiting plant litter, yielding numerous new findings (Liu et al. 2015; Tanaka et al. 2015; Hyde et al. 2016; Hashimoto et al. 2017; Crous et al. 2020; Tennakoon et al. 2024). Particularly, we are exploring the hidden diversity of Dothideomycetes fungi in plant litter through comprehensive morphology and phylogenetic analyses (Tennakoon et al. 2018, 2020, 2023, 2024; Hyde et al. 2020; Hongsanan et al. 2020; Wanasinghe et al. 2022).

Acrocalymmaceae and Didymosphaeriaceae are two of the speciose families in the Pleosporales (Dothideomycetes, Ascomycota). Of them, Acrocalymmaceae was established by Trakunyingcharoen et al. (2014) to include the monotypic genus *Acrocalymma* with *A. medicaginis* as the type species. Most of the Acrocalymmaceae species have been reported in their asexual morph, characterized by immersed to semi-immersed, globose to sub-globose conidiomata and cylindrical to fusiform, hyaline or pale brown, aseptate, or 1–3-septate conidia with appendages at both ends (Hongsanan et al. 2020; Mortimer et al. 2021; Calabon et al. 2023). The sexual morph has immersed to erumpent, globose to sub-globose, unilocular ascomata, cylindrical asci, and narrowly fusiform, hyaline to brown or pale brown, 1–3-septate ascospores with a mucoid sheath (Jayasiri et al. 2019; Mortimer et al. 2021; Tennakoon et al. 2021; Konta et al. 2023). They have been reported from worldwide, including temperate, subtropical, and tropical regions (e.g., Australia, China, Egypt, India, Spain, Thailand, and the United States) (Shoemaker et al. 1991; Trakunyingcharoen et al. 2014; Dong et al. 2020; Tennakoon et al. 2021; Calabon et al. 2023; Konta et al. 2023). Most species have been found as saprobes on plant litter, which includes dead leaves, wood, pods, and seeds, in both terrestrial and freshwater habitats (Jayasiri et al. 2019; Mortimer et al. 2021; Tennakoon et al. 2021). Some species (e.g., *A. medicaginis* and *A. vagum*) have also been confirmed as plant pathogens (Alcorn and Irwin 1987; Farr et al. 1998; Trakunyingcharoen et al. 2014). Additionally, they have been reported as endophytes, for instance, *A. vagum* from wild rice (Zeng et al. 2023).

Didymosphaeriaceae, introduced by Munk (1956), is typified by the genus *Didymosphaeria*, and *D. epidermidis* is the type species. The morphological characteristics of Didymosphaeriaceae are highly diverse, with most sexual morphs having 1–3-septa (e.g., *Barria*, *Didymosphaeria*, *Kalmusia*, *Letendraea*, and *Neokalmusia*) or muriform (e.g., *Austropleospora*, *Bimuria*, *Deniquelata*, *Julella*, *Karstenula*, and *Tremateia*), brown ascospores and trabeculate pseudoparaphyses that are anastomosed above the asci (Ariyawansa et al. 2014a, b; Liu et al. 2015; Hongsanan et al. 2020). Asexual morphs can be either coelomycetes (e.g., *Alloconiothyrium*, *Austropleospora*, *Letendraea*, *Paraconiothyrium*, and *Paraphaeosphaeria*) or hyphomycetes (e.g., *Pseudopithomyces*, and *Spegazzinia*) (Hyde et al. 2013; Wanasinghe et al. 2016; Hongsanan et al. 2020). The ordinal placement of Didymosphaeriaceae has been a subject of controversy for a long time. Initially, this was placed in Melanommatales based on trabeculate pseudoparaphyses (Barr 1990). Subsequently, Ariyawansa et al. (2014a) provided a comprehensive taxonomic revision of this group, revealing that the family appears to be a distinct family within Pleosporales. Most of the Didymosphaeriaceae species have been recognized as saprobes in terrestrial, freshwater, marine, and mangrove ecosystems (Ariyawansa et al. 2014a, b; Liu et al. 2015; Phookamsak et al. 2019; Tennakoon et al. 2022). Additionally, some taxa have been reported as both endophytes and pathogens in plants (Yuan et al. 2020; Guarnaccia et al. 2022). Currently, there are 33 accepted genera in this family (Wijayawardene et al. 2022, Htet et al. 2024).

During a mycological survey conducted in China, we isolated seven saprobic fungi from plant litter substrates. The objectives of this study are to identify the isolated taxa using both morphological and phylogenetic approaches. Our findings contribute to the growing body of knowledge on fungal diversity in China.

## ﻿Materials and methods

### ﻿Sample collection and examination

Dead wood and leaf specimens were collected from Guangdong and Yunnan Provinces, China (during 2017–2024). The collected specimens were placed in zip-lock bags with collection information (Rathnayaka et al. 2025), brought to the laboratory, and incubated in a plastic box with wet tissue paper (two days). The specimens were initially checked using a stereomicroscope (AXIOSKOP 2 PLUS Series, Göttingen, Germany). All the surface characteristics of ascomata and conidiomata, such as color, shape, and position of the host surface (e.g., immersed, semi-immersed, and erumpent or superficial), were recorded. Hand sections of ascomata and conidiomata were obtained manually using a razor blade and were mounted in distilled water. Micro-morphological characteristics (e.g., ascospores, asci, conidia, conidiogenous cells, peridium, pseudoparaphyses) were examined and photographed using a Nikon ECLIPSE Ni-U compound microscope (Nikon Corporation, Japan), equipped with a Canon Axiocam 506 color digital camera (Hanover, Germany). Lactoglycerol was used to prepare the permanent slides, and nail polish was applied to the margins of the cover slips to seal them. All measurements were calculated using the Tarosoft (R) Image Framework application. Adobe Photoshop CS3 Extended version 10.0 software (Adobe Systems, USA) was used to construct the photo plates.

The approach described by Senanayake et al. (2022) was used for single-spore isolation. After germination, spores were carefully transferred to Potato Dextrose Agar (PDA) and incubated at 25 °C. Pure cultures were obtained after a subsequent subculturing process. Culture characteristics (on PDA) were observed after three weeks. Type specimens were deposited in the Herbarium of Fungi, Shenzhen University, Shenzhen, China. Living cultures were deposited in the Culture Collection of Microbial Shenzhen University (MBSZU), Shenzhen University, Shenzhen, China. According to Jayasiri et al. (2015) and Index Fungorum (2025), the Faces of Fungi (FOF) and Index Fungorum (IF) numbers were obtained.

### ﻿DNA extraction, PCR amplification, and sequencing

Fungal cultures grown on PDA for 4 weeks at 25 °C were used for DNA extraction. Axenic mycelium (50–100 mg) was scraped from the growing cultures. The extraction process was performed using a DNA extraction kit (E.Z.N.A. Fungal DNA Mini Kit, D3390-02, Omega Bio-Tek) according to the manufacturer’s protocol. In the case of *Acrocalymma ampeli*, *A. poaceicola*, and *Neokalmusia arundinis* (their spores were not germinated), DNA was isolated directly from fungal fruiting bodies on their natural substrate using a commercial DNA extraction kit (E.Z.N.A. ® Forensic DNA Kit, D3591-01, Omega BIO-TEK). The DNA products were kept at 4 °C, and replicate samples were stored at -20 °C to ensure long-term stability. The following genomic regions were amplified: the internal transcribed spacer (ITS1–5.8S–ITS2), 28S large subunit rDNA (LSU), 18S small subunit rDNA (SSU), translation elongation factor 1-alpha gene (*tef*1-α), and beta-tubulin (*tub*2). The ITS region was amplified using primers ITS4 and ITS5 (White et al. 1990), the LSU region with LR0R and LR5 (Vilgalys and Hester 1990), and the SSU region with NS1 and NS4 (White et al. 1990). The *tef*1-α gene was amplified using EF1-983F and EF1-2218R (Rehner 2001), and *tub*2 was amplified with Bt2a/Bt2b (Glass and Donaldson 1995). The total volume (25 µl) of the PCR reaction contained 9.5 µl of sterilized distilled water, 12.5 µl of 2 × Power Taq PCR MasterMix (a premix and ready-to-use solution, including 0.1 Units/μl Taq DNA Polymerase and 500 μM dNTP Mixture each (dATP, dCTP, dGTP, dTTP) (Bioteke Co., China), 1 μl of each forward and reverse primer (stock concentration 10 pM), and 1 μl of DNA template. Thermal cycle programs for LSU, SSU, ITS, *tef*1-α, and *tub*2 genes were followed as mentioned in Tennakoon et al. (2021) and Rathnayaka et al. (2023). The PCR products were quantified using a NanoDrop One spectrophotometer (Thermo Fisher Scientific, USA), verified by 1% agarose gel electrophoresis, and subsequently sequenced by Sangon Biotech (Shanghai) using the original PCR primers.

### ﻿Phylogenetic analyses

The obtained sequences (forward and reverse) were initially checked with BioEdit v 7.0.5.3 (Hall 1999) and assembled using SeqMan v. 7.0.0 (DNASTAR, Madison, WI). A nucleotide BLAST search (https://blast.ncbi.nlm.nih.gov/) was conducted to identify strains with high similarity. Additional related sequences were obtained from recently published data (Tennakoon et al. 2022; Calabon et al. 2023; Konta et al. 2023; Htet et al. 2024). All single-gene sequence datasets were aligned using MAFFT version 7 (Katoh and Standley 2013) through its online platform (https://mafft.cbrc.jp/alignment/software/). Single-gene alignment matrices were trimmed to remove ambiguous bases using TrimAl 1.2 (Capella-Gutiérrez et al. 2009). The aligned sequences were concatenated using BioEdit version 7.2.5 (Hall 1999).

The concatenated alignment was subjected to phylogenetic analysis using both Maximum Likelihood (ML) and Bayesian Inference (BI) approaches. Substitution models were selected using MrModeltest v. 2.3 (Nylander 2004) based on the Akaike information criterion (AIC). Maximum Likelihood analysis was performed using RAxML-HPC2 v8.2.8 (Stamatakis 2014) on the CIPRES Science Gateway (Miller et al. 2010), implementing the GTR+I+G model of nucleotide substitution. Bayesian Inference was performed using MrBayes v3.2.1 (Ronquist et al. 2012) with six simultaneous Markov chains run for 3 million generations, sampling trees every 100^th^ generation. The initial 20% of sampled trees were discarded as burn-in, with the remaining 80% used to calculate posterior probabilities and construct the consensus tree. Phylogenetic trees were visualized and initially edited using FigTree v1.4.0 (Rambaut 2012), with final graphical adjustments and layout preparation performed in Microsoft PowerPoint 2010 (Microsoft Corp.) and Adobe Illustrator CS3 (v15.0.0, Adobe Inc.). The newly generated sequences were deposited in GenBank (accession numbers provided in Tables 1–3).

**Table 1.** Taxa used in the phylogenetic analyses of Acrocalymmaceae and their corresponding GenBank numbers of partial ITS, LSU, and SSU sequences. Isolates/sequences in bold were isolated/sequenced in the present study. Unavailable sequences are indicated as “NA”.

Taxon	Strain / voucher number	GenBank Accession Numbers		
		SSU	LSU	ITS
*Acrocalymma ampeli*	MFLUCC 20-0159	MW079341	MW063211	MW063150
*A. ampeli*	NCYUCC 19-0288	MW079342	MW063212	MW063151
***A. ampeli***	**SZU25-013**	**PV730035**	**PV730024**	**PV730045**
***A. ampeli***	**SZU25-014**	**PV730036**	**PV730025**	**PV730046**
*A. aquaticum*	MFLUCC 11-0208	JX276953	NG_042698	NR_121544
*A. aquaticum*	CC36/MFLUCC 20-0124	NA	MT875393	MT875395
*A. arengae*	MFLUCC 15–0327A	ON650177	ON650673	ON650154
*A. arengae*	MFLUCC 15–0327B	ON650178	ON650674	ON650155
*A. bilobatum*	K.L. Chen L119	NA	NA	KX034339
A. bilobatum*	MFLUCC 20-0125	NA	MT875394	MT875396
*A. bipolare*	MD1321	NA	NG_075326	NA
*A. chuxiongense*	IFRDCC3104	NA	ON596248	ON595715
*A. cycadis*	CBS 137972	NA	NG_057046	NR_137884
*A. fici*	CBS 317.76	NA	NG_057056	NR_137953
*A. fici*	MFLUCC 21-0103	NA	MT860429	MT864351
*A. guizhouense*	CGMCC 3.20853	OM838471	OM838474	OM838410
*A. guizhouense*	GZUIFR H22.028	OM838472	OM838475	OM838411
*A. guizhouense*	GZUIFR H22.029	OM838473	OM838476	OM838412
*A. hongheense*	HKAS 111907	MW424792	MW424777	MW424763
*A. hongheense*	HKAS 111908	MW424791	MW424776	MW424762
*A. hongheense*	HKAS 111909	MW424790	MW424775	MW424761
*A. magnoliae*	MFLUCC 18–0545	OL331094	OK655819	OL413439
*A. magnoliae*	MFLUCC 21–0204	OL331095	OK655820	OL413440
*A. medicaginis*	CPC 24340	NA	KP170713	KP170620
*A. medicaginis*	MFLUCC 17-1423	MT214387	MT214432	MT214338
*A. medicaginis*	MFLUCC 17-1439	MT214388	MT214433	MT214339
*A. pterocarpi*	MFLUCC 17-0926	MK347840	NG_066306	MK347732
*A. pterocarpi*	MFLUCC 18–0718	OL331093	OK655818	OL413438
*A. pterocarpi*	NC13-171	NA	LC517881	LC517880
*A. paeoniae*	CGMCC:3.24440	OR253217	OR253308	OR253149
***A. poaceicola***	**SZU25-015**	**PV730032**	**PV730021**	**PV730042**
***A. poaceicola***	**SZU25-016**	**PV730033**	**PV730022**	**PV730043**
***A. poaceicola***	**SZU25-017**	**PV730034**	**PV730023**	**PV730044**
*A. vagum*	CPC 24225	NA	NA	KP170635
*A. vagum*	CPC 24226	NA	NA	KP170636
*A. walkeri*	UTHSC DI16-195	NA	LN907338	LT796832
*A. yuxiense*	HKAS 111910	MW424793	MW424778	NA
*Ascocylindrica marina*	MD6011	KT252907	KT252905	NA
*Ascocylindrica marina*	MF416	MK007124	MK007123	NA
*Boeremia exigua*	CBS 431.74	EU754084	EU754183	FJ427001
*Boeremia foveata*	CBS 341.67	GU238203	GU237947	GU237834

**Table 2.** Taxa used in the phylogenetic analyses of Didymosphaeriaceae and their corresponding GenBank numbers of partial ITS, LSU, SSU, and *tef1-α* sequences. Isolates/sequences in bold were isolated/sequenced in the present study. Unavailable sequences are indicated as “NA”.

Taxon	Strain / voucher number	GenBank Accession Numbers			
		LSU	SSU	ITS	tef 1-α
*Alloconiothyrium aptrootii*	CBS 980.95	JX496234	NA	JX496121	NA
*A. aptrootii*	CBS 981.95	JX496235	NA	JX496122	NA
* encephalarti *	CPC 35980	MN567610	NA	MN562102	NA
*Kalmusia erioi*	MFLU 18-0832	MN473052	MN473046	MN473058	MN481599
*K. italica*	MFLU 14-0620	KP325441	KP325442	KP325440	NA
*Laburnicola muriformis*	MFLUCC 14-0921	KU743201	KU743202	KU743200	NA
*L. muriformis*	MFLUCC 16-0290	KU743198	KU743199	KU743197	NA
*L. muriformis*	MFLUCC 14-0921	KU743201	KU743202	KU743200	NA
*L. muriformis*	MFLUCC 16-0290	KU743198	KU743199	KU743197	NA
*Neokalmusia arundinis*	MFLU 17-0754	MT649878	MT649880	MT649882	MT663766
*N. arundinis*	MFLUCC 15-0463	NG_068237	NG_068372	NR_165852	KY244024
*N. arundinis*	MFLUCC 14-0222	KX954400	KX986344	KX965731	KY271091
***N. arundinis***	**SZU25-019**	**PV730028**	**PV730039**	**PV730049**	**PV749905**
*N. brevispora*	KT 2313	AB524601	AB524460	LC014574	AB539113
*N. brevispora*	KT 1466	AB524600	AB524459	LC014573	AB539112
*N. didymospora*	MFLUCC 11-0613	KP091434	KP091435	NA	NA
*N. jonahhulmei*	KUMCC 21-0819	ON007040	ON007049	ON007044	ON009134
*N. kunmingensis*	KUMCC 18-0120	MK079889	MK079887	MK079886	MK070172
*N. scabrispora*	KT 1023	AB524593	AB524452	LC014575	AB539106
*N. scabrispora*	KT 2202	AB524594	AB524453	LC014576	AB539107
*N. thailandica*	MFLUCC 16-0405	NG_059792	KY706137	NR_154255	KY706145
*Neptunomyces aureus*	CMG11	NA	NA	MK912120	MK947999
*Ne. aureus*	CMG12	NA	NA	MK912121	MK948000
*Ne. aureus*	CMG13	NA	NA	MK912122	MK948001
*Ne. aureus*	CMG14	NA	NA	MK912123	MK948002
***N.. chinensis***	**SZU25-020**	**PV730026**	**PV730037**	**PV730047**	**PV749903**
***N.. chinensis***	**SZU25-021**	**PV730027**	**PV730038**	**PV730048**	**PV749904**
*N.. jeanbriggsiae*	BRIP 75897a	PQ431200	NA	PQ431208	NA
*Ne. juncicola*	CPC 45436	PP791464	NA	PP791436	PP780627
*N.. litoralis*	BRIP 75555a	OR259052	NA	OR271911	NA
*N.. soli*	MFLUCC 24-0272	PQ306558	NA	NA	NA
*N.. soli*	MFLUCC 24-0279	PQ306560	NA	PQ306559	NA
*Pseudopithomyces atro-olivaceus*	CBS 244.96	LT671616	NA	LT671625	NA
*P. chartarum*	C459	MK348027	MK347916	MK347808	NA
*P. chartarum*	C449	MK348017	MK347906	MK347798	NA
*P. chartarum*	UTHSC 03-2472	HG518064	NA	HG518059	NA
*P. chartarum*	MFLUCC 17-0314	MF173605	MF173606	MF173607	NA
*P. chartarum*	C284	MK347964	MK347854	MK347747	NA
*P. chartarum*	C447	MK348015	MK347904	MK347796	NA
***P. chartarum***	**SZU25-022**	**PV730030**	**PV730040**	**PV730051**	**PV749906**
*P. diversisporus*	UTHSC 06-4528	HG933827	NA	HG933810	NA
*P. entadae*	C227	MK347946	MK347837	NA	NA
*P. karoo*	CBS 804.72	NG_057865	HG933811	NR_154291	NA
*P. maydicus*	MFLUCC 14-0391	KX034666	NA	KX034668	NA
*P. maydicus*	MFLUCC 17-0028	NA	NA	MG545071	NA
*P. palmicola*	MFLUCC 14-0392	KX034665	NA	NR_154345	NA
*P. sacchari*	CBS 803.72	MH872301	LK936379	NA	NA
*Spegazzinia sp.*	yone 279	AB807583	AB797293	NA	AB808559
*Spegazzinia sp.*	CL115	AY234948	NA	NA	NA
*S. bromeliacearum*	URM 8084	MK809513	NA	MK804501	
*S. cameliae*	CMU 328	MH734521	MH734523	MH734522	MH734524
*S. deightonii*	yone 212	AB807582	AB797292	NA	AB808558
*S. deightonii*	MFLUCC 20-0002	MN956772	MN956770	MN956768	MN927133
*S. deightonii*	yone 66	AB807581	AB797291	NA	AB808557
*S. deightonii*	MFLUCC 18-1625	ON117309	ON117273	ON117291	ON158097
***S. deightonii***	**SZU25-023**	**PV730031**	**PV730041**	**PV730052**	**PV749907**
*S. intermedia*	CBS 249.89	MH873861	NA	MH862171	NA
*S. lobulata*	CBS 361.58	MH869344	NA	MH857812	NA
*S. musae*	MFLUCC 20-0001	MN930514	MN930513	MN930512	MN927132
*S. neosundara*	MFLUCC 15–0456	KX954397	KX986341	KX965728	NA
*S. neosundara*	MFLUCC 13-0211	MH040812	MH040811	MH040810	MH055460
*S. tessarthra*	SH 287	AB807584	AB797294	NA	AB808560
*S. tessarthra*	NRRL 54913	NA	NA	JQ673429	NA
*S. tessarthra*	ASV319	NA	NA	MN898233	NA
*S. tessarthra*	MFLUCC 17-2249	MH071197	MH071192	MH071193	NA
*S. tessarthra*	12H0104	NA	NA	KT385776	NA
*S. tessarthra*	MFLUCC 18-1624	ON117308	ON117272	ON117290	ON158096
*S. radermacherae*	C264	MK347957	MK347848	MK347740	NA
*Xenocamarosporium acaciae*	ZHKUCC:22-0202	OP297775	OP297789	OP297805	OP321574
*X. acaciae*	ZHKUCC:22-0203	OP297776	OP297790	OP297806	OP321575

**Table 3.** Taxa used in the phylogenetic analyses of *Paraconiothyrium* and their corresponding GenBank numbers of partial ITS, LSU, and *tub*2 sequences. Isolates/sequences in bold were isolated/sequenced in the present study. Unavailable sequences are indicated as “NA”.

Species	Strain/Voucher No.	GenBank Accession No.		
		LSU	ITS	tub2
*Paraconiothyrium ajrekarii*	NFCCI 4810	MT372905	MT372906	MT394161
*P. archidendri*	CBS 168.77	MH872813	MH861045	JX496388
*P. archidendri*	964-SAB SA1 3	NA	MT820342	NA
*P. archidendri*	C321	MK347974	MK347757	NA
*P. archidendri*	1-3-10-2-1-4	NA	KX065269	NA
*P. archidendri*	NNIBRFG116	NA	KY327413	NA
*P. archidendri*	NNIBRFG99	NA	KY327412	NA
*P. archidendri*	NNIBRFG29	NA	KY327411	NA
*P. archidendri*	MFLUCC 19-0043	ON117302	ON117284	NA
***P. archidendri***	**SZU25-018**	**PV730029**	**PV730050**	**PV749908**
*P. babiogorense*	CBS 128292	MH876291	MH864845	NA
*P. brasiliense*	CBS 115.92	JX496135	JX496022	JX496361
*P. brasiliense*	CBS 395.87	JX496196	JX496083	JX496422
*P. brasiliense*	CBS 122320	JX496146	JX496033	JX496372
*P. brasiliense*	CBS 122851	JX496149	JX496036	JX496375
*P. brasiliense*	MFLUCC 19-0040	ON117301	ON117283	NA
*P. camelliae*	NTUCC 18-096	MT071269	MT112293	MT308623
*P. cyclothyrioides*	CBS 972.95	JX496232	JX496119	JX496458
*P. cyclothyrioides*	CBS 432.75	JX496201	JX496088	JX496427
*P. cyclothyrioides*	SN 3169-19	NA	MN416682	NA
*P. cyclothyrioides*	NNIBRFG3266	MW237682	NA	NA
*P. cyclothyrioides*	NNIBRFG3255	MW237679	NA	NA
*P. cyclothyrioides*	EXF-14614	NA	MT280696	NA
*P. cyclothyrioides*	NFCCI 4387	MN241143	MN242780	NA
*P. estuarinum*	CBS 109850	JX496129	JX496016	JX496355
*P. fuckelii*	JZB320001	MN519513	MN495986	MN508193
*P. fuckelii*	MFLUCC 13-0073	KJ939281	KJ939278	NA
*P. fuckelii*	JZB320002	MN519514	MN495987	MN508194
*P. fuckelii*	JZB320003	MN519515	MN495988	MN508195
*P. fuckelii*	JZB320004	MN519516	MN495989	MN508196
*P. fuckelii*	CBS 584.69	NA	JX496211	JX496437
*P. fuckelii*	CVG970	NA	MZ712975	NA
*P. fuckelii*	CBS 508.94	JX496209	JX496096	JX496435
*P. fuckelii*	CBS 653.85	JX496217	JX496104	JX496443
*P. fuckelii*	CBS 764.71B	JX496225	JX496112	JX496451
*P. fuckelii*	MFLUCC 19-0067	ON117305	ON117287	NA
*P. fuckelii*	CBS 797.95	JX496226	JX496113	JX496452
*P. fuscomaculans*	CBS 116.16	MH866170	MH854649	–
*P. hakeae*	CBS 142521	KY979809	KY979754	KY979920
*P. iridis*	CBS:146036	MT223919	MT223827	MT223743
*P. lini*	CBS 253.92	GU238093	NA	KT266268
*P. lycopodinum*	CBS 134705	MH877564	NA	NA
*P. maculicutis*	CBS 101461	EU754200	NA	NA
*P. magnoliae*	MFLUCC 10-0278	KJ939283	KJ939280	NA
*P. nelloi*	MFLU 14-0813	KP711365	KP711360	NA
*P. polonense*	CBS 134153	KF700360	NA	NA
*P. rosae*	MFLU 15-1115	MG829041	MG828932	NA
*P. thysanolaenae*	MFLUCC 10-0550	KP744496	KP744453	NA
*P. tiliae*	CBS 265.94	EU754139	NA	NA
*P. zingiberacearum*	MFLUCC 18-0559	ON117303	ON117285	ON158098
*P. zingiberacearum*	NCYUCC 19-0230	ON117304	ON117286	ON158099
*Tremateia arundicola*	MFLU 16-1275	KX274248	KX274241	NA
*Tremateia guiyangensis*	GZAAS01	KX274247	KX274240	NA

## ﻿Results

### ﻿Phylogenetic analyses

**Analysis 1 – Acrocalymmaceae**

**Figure 1.**
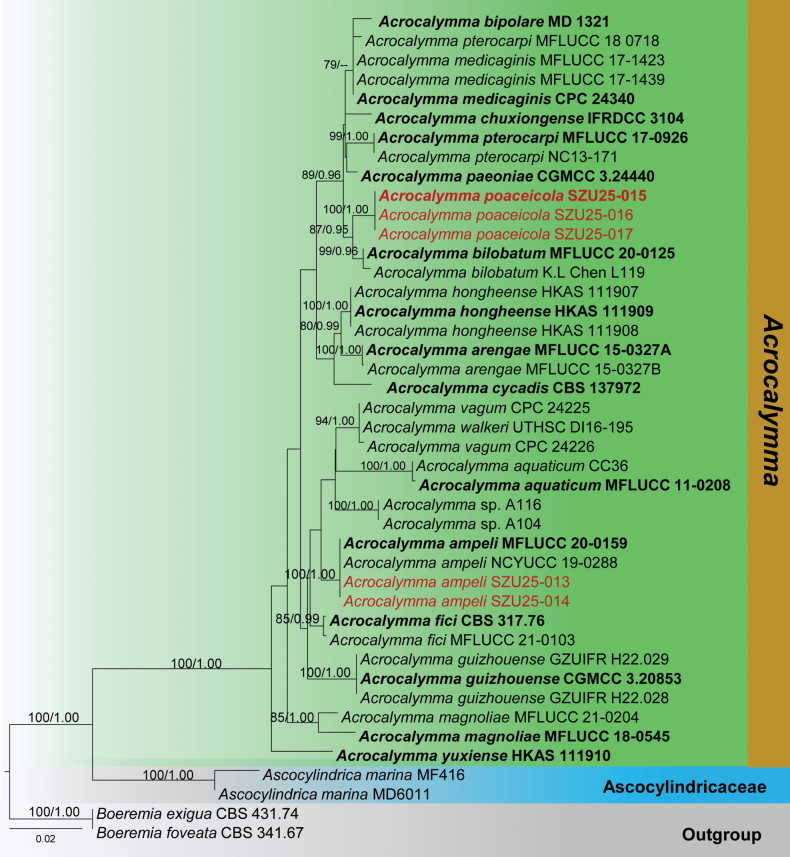
Phylogram generated from Maximum Likelihood analysis is based on combined LSU, SSU, and ITS sequence data. The tree is rooted with *Boeremia exigua* (CBS 431.74) and *B. foveata* (CBS 341.67). The new isolates are in red, and ex-type strains are indicated in bold face. Bootstrap support values ≥ 65% from the Maximum Likelihood (ML) and Bayesian posterior probabilities (BYPP) values ≥ 0.90 are indicated above the nodes, respectively.

The combined LSU-SSU-ITS dataset contained 2818 characters, including gaps. *Boeremia exigua* (CBS 431.74) and *B. foveata* (CBS 341.67) were used as outgroup taxa. The RAxML analysis of the combined dataset yielded a best-scoring tree (Fig. 1). The final ML optimization likelihood value was -7357.640966. There were 29.71% undetermined characters or gaps and 509 distinct alignment patterns. Estimated base frequencies were A = 0.250436, C = 0.216202, G = 0.272118, T = 0.261245; substitution rates AC = 1.640471, AG = 1.667992, AT = 2.184081, CG = 0.699183, CT = 5.878108, GT = 1.000; proportion of invariable sites I = 0.692125; gamma distribution shape parameter *α* = 0.639926. The Bayesian analysis has resulted in 30,000 trees after 3,000,000 generations. Bootstrap support values for ML higher than 70% and BYPP greater than 0.90 are given above each branch, respectively (Fig. 1). All analyses (ML and BYPP) yielded similar topologies and concurred with previous studies (Calabon et al. 2023; Konta et al. 2023).

According to the phylogeny, our collection (SZU25-015, SZU25-016, and SZU25-017) clusters within the *Acrocalymma* species. New isolates cluster together and show an independent lineage sister to *A. bilobatum* isolates (MFLUCC 20-0125 and KL Chen L119) with robust statistical support (87% ML, 0.95 BYPP). In addition, the isolates SZU25-013 and SZU25-014 cluster with *A. ampeli* isoates (MFLU 19-2734 and NCYU 19-0008) in a monophyletic clade (100% ML, 1.00 BYPP).

**Analysis 2 – *Neokalmusia* and *Neptunomyces***

**Figure 2.**
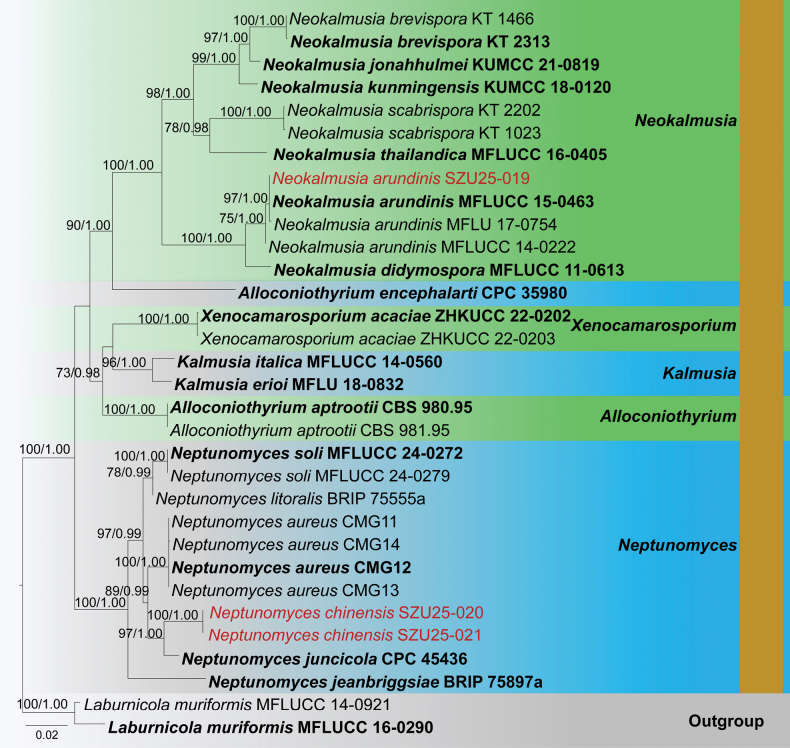
Phylogram generated from the Maximum Likelihood analysis is based on combined LSU, SSU, ITS, and *tef*1-α sequence data. The tree is rooted with *Laburnicola muriformis* (MFLUCC 14-0921 and MFLUCC 16-0290). The new isolates are in red, and ex-type strains are indicated in bold face. Bootstrap support values ≥ 65% from the Maximum Likelihood (ML) and Bayesian posterior probabilities (BYPP) values ≥ 0.90 are indicated above the nodes, respectively.

The combined LSU-SSU-ITS-*tef*1-α dataset contained 3649 characters, including gaps. *Laburnicola muriformis* (MFLUCC 14-0921 and MFLUCC 16-0290) was used as the outgroup taxon. The RAxML analysis of the combined dataset yielded a best-scoring tree (Fig. 2). The final ML optimization likelihood value was -11623.840913. There were 32.16% undetermined characters or gaps and 800 distinct alignment patterns. Estimated base frequencies were A = 0.233832, C = 0.260249, G = 0.273224, T = 0.232696; substitution rates AC = 1.154239, AG = 1.660022, AT = 1.406193, CG = 1.070701, CT = 6.334516, GT = 1.000; proportion of invariable sites I = 0.591573; gamma distribution shape parameter *α* = 0.570976. The Bayesian analysis has resulted in 30,000 trees after 3,000,000 generations. Bootstrap support values for ML higher than 70% and BYPP greater than 0.90 are given above each branch, respectively (Fig. 2). All analyses (ML and BYPP) yielded similar topologies and concurred with previous studies (Crous et al. 2024; Yasanthika et al. 2024).

Phylogeny indicates that our collection (SZU25-020 and SZU25-021) clusters within *Neptunomyces* species. Both isolates cluster together and show an independent lineage sister to *N. juncicola* (CPC 45436) with robust statistical support (97% ML, 1.00 BYPP). In addition, the isolate SZU25-019 clusters with *Neokalmusia arundinis* isolates (MFLUCC 15-0463, MFLU 17-0754, and MFLUCC 14-0222) in a monophyletic clade (75% ML, 1.00 BYPP).

**Analysis 3 – *Paraconiothyrium***

**Figure 3.**
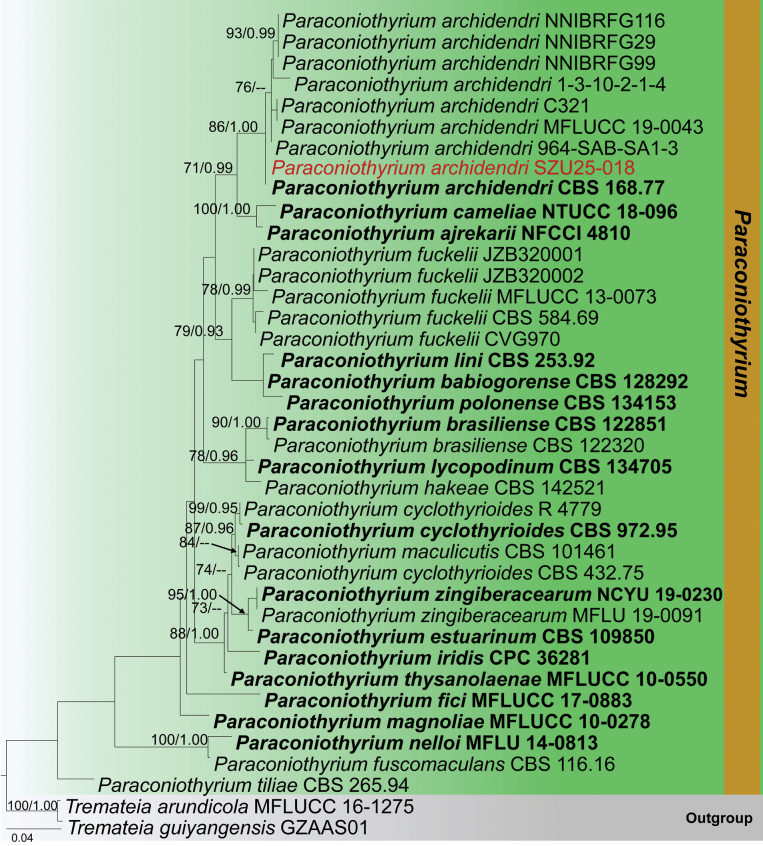
Phylogram generated from the Maximum Likelihood analysis is based on combined LSU, ITS, and *tub*2 sequence data. The tree is rooted with *Tremateia arundicola* (MFLUCC 16-1275) and *T. guiyangensis* (GZAAS01). The new isolates are in red, and ex-type strains are indicated in bold face. Bootstrap support values ≥ 65% from the Maximum Likelihood (ML) and Bayesian posterior probabilities (BYPP) values ≥ 0.90 are indicated above the nodes, respectively.

The combined LSU-ITS-*tub2* dataset contained 2145 characters, including gaps. *Tremateia arundicola* (MFLUCC 16-1275) and *T. guiyangensis* (GZAAS01) were used as outgroup taxa. The RAxML analysis of the combined dataset yielded the best-scoring tree (Fig. 3). The final ML optimization likelihood value was -7286.035715. There were 35.11% undetermined characters or gaps and 649 distinct alignment patterns. Estimated base frequencies were A = 0.230153, C = 0.249594, G = 0.282309, T = 0.237944; substitution rates AC = 1.589743, AG = 2.770684, AT = 1.318375, CG = 0.871420, CT = 6.249278, GT = 1.000; proportion of invariable sites I = 0.566479; gamma distribution shape parameter *α* = 0.550172. The Bayesian analysis has resulted in 30,000 trees after 3,000,000 generations. Bootstrap support values for ML higher than 70% and BYPP greater than 0.90 are given above each branch, respectively (Fig. 3). All analyses (ML and BYPP) yielded similar topologies and concurred with previous studies (Guarnaccia et al. 2022; Tennakoon et al. 2022). Phylogeny indicates that our collection (SZU25-018) clusters within *Paraconiothyrium archidendri* isolates (MFLUCC 19-0043, 964-SAB-SA1-3, CBS 168.77, and C321) in a well-supported clade (86% ML, 1.00 BYPP).

**Analysis 4 – *Pseudopithomyces***

**Figure 4.**
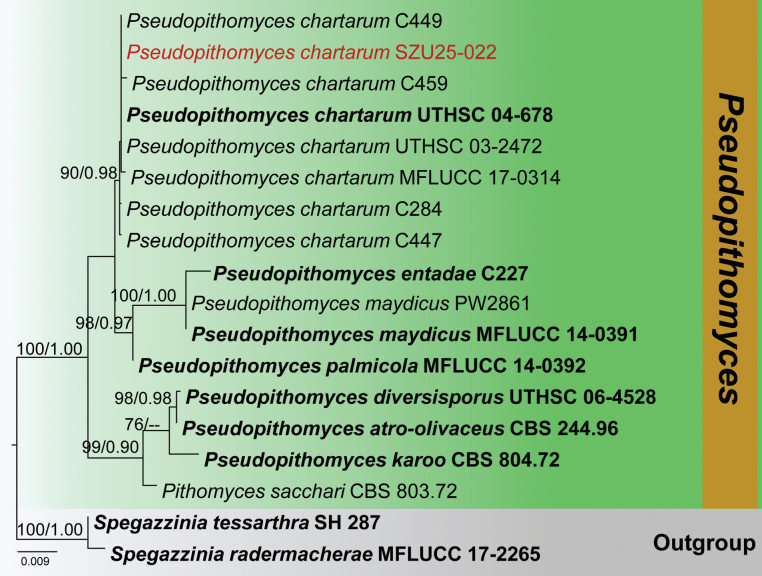
Phylogram generated from the Maximum Likelihood analysis is based on combined LSU, SSU, ITS, and *tef*1-α sequence data. The tree is rooted with *Spegazzinia radermacherae* (MFLUCC 17-2265) and *S. tessarthra* (SH 287). The new isolates are in red, and ex-type strains are indicated in bold face. Bootstrap support values ≥ 65% from the Maximum Likelihood (ML) and Bayesian posterior probabilities (BYPP) values ≥ 0.90 are indicated above the nodes, respectively.

The combined LSU-SSU-ITS-*tef*1-α dataset comprised 3,215 characters, including gaps. *Spegazzinia radermacherae* (MFLUCC 17-2265) and *S. tessarthra* (SH 287) were used as outgroup taxa. The RAxML analysis of the combined dataset yielded the best-scoring tree (Fig. 4). The final ML optimization likelihood value was -6160.156800. There were 41.67% undetermined characters or gaps and 243 distinct alignment patterns. Estimated base frequencies were A = 0.237009, C = 0.251568, G = 0.272682, T = 0.238741; substitution rates AC = 1.586697, AG = 1.677838, AT = 1.298195, CG = 1.029806, CT = 5.817290, GT = 1.000; proportion of invariable sites I = 0.572938; gamma distribution shape parameter *α* = 0.783990. The Bayesian analysis has resulted in 30,000 trees after 3,000,000 generations. Bootstrap support values for ML higher than 70% and BYPP greater than 0.90 are given above each branch, respectively (Fig. 4). All analyses (ML and BYPP) yielded similar topologies and concurred with previous studies (Samaradiwakara et al. 2022; Wu et al. 2023). Phylogeny indicates that our collection (SZU25-022) clusters within *Pseudopithomyces chartarum* isolates (C284, C447, C449, C459, UTHSC 04-678, UTHSC 03-2472, and MFLUCC 17-0314) in a well-supported clade (90% ML, 0.98 BYPP).

**Analysis 5 – *Spegazzinia***

**Figure 5.**
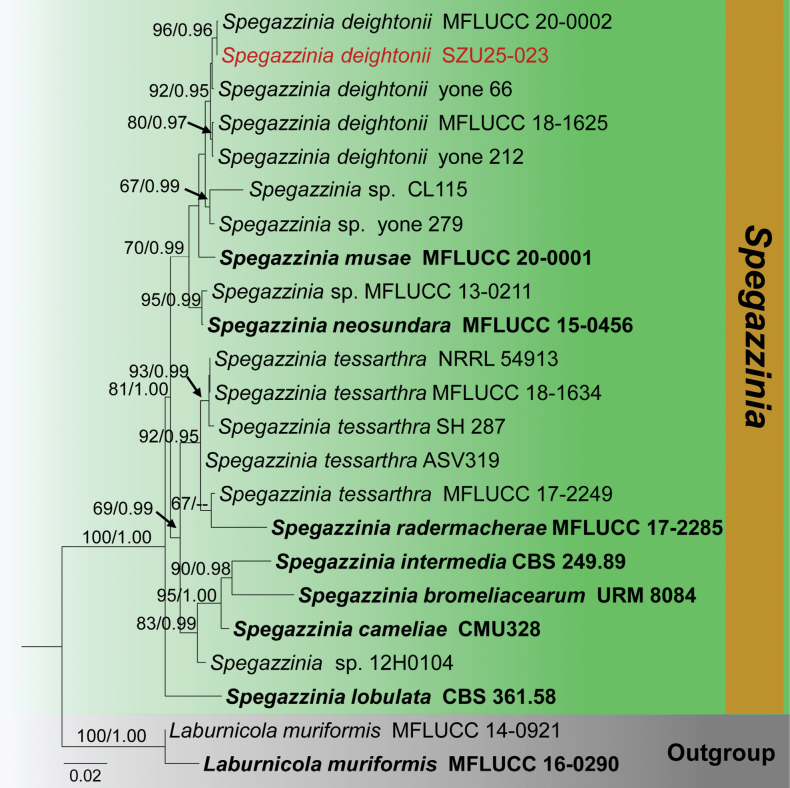
Phylogram generated from the Maximum Likelihood analysis is based on combined LSU, SSU, ITS, and *tef*1-α sequence data. The tree is rooted with *Laburnicola muriformis* (MFLUCC 14-0921 and MFLUCC 16-0290). The new isolates are in red, and ex-type strains are indicated in bold face. Bootstrap support values ≥ 65% from the Maximum Likelihood (ML) and Bayesian posterior probabilities (BYPP) values ≥ 0.90 are indicated above the nodes, respectively.

The combined LSU-SSU-ITS-*tef1-α* dataset contained 3495 characters, including gaps. *Laburnicola muriformis* (MFLUCC 14-0921 and MFLUCC 16-0290) were used as outgroup taxa. The RAxML analysis of the combined dataset yielded the best-scoring tree (Fig. 5). The final ML optimization likelihood value was -8278.609544. There were 35.24% undetermined characters or gaps and 553 distinct alignment patterns. Estimated base frequencies were A = 0.238139, C = 0.257164, G = 0.274729, T = 0.229967; substitution rates AC = 1.482308, AG = 2.141805, AT = 1.664137, CG = 1.084752, CT = 6.606857, GT = 1.000; proportion of invariable sites I = 0.670885; gamma distribution shape parameter *α* = 0.650417. The Bayesian analysis has resulted in 30,000 trees after 3,000,000 generations. Bootstrap support values for ML higher than 70% and BYPP greater than 0.90 are given above each branch, respectively (Fig. 5). All analyses (ML and BYPP) yielded similar topologies and concurred with previous studies (Samarakoon et al. 2020; Tennakoon et al. 2022). Phylogeny indicates that our collection SZU25-023 clusters within *Spegazzinia deightonii* isolates (MFLUCC 20-0002, MFLUCC 18-1625, yone 66, and yone 212) in a well-supported clade (92% ML, 0.95 BYPP).

## ﻿Taxonomy

### ﻿ Acrocalymma

Taxon classificationFungiPleosporalesDidymosphaeriaceae

Alcorn & J.A.G. Irwin, Trans. Br. mycol. Soc. 88(2): 163 (1987)

9463BE71-AA31-56FF-9BA9-32830A3E5A7F

#### Notes.

Alcorn and Irwin (1987) introduced *Acrocalymma* to include *A. medicaginis*, which was recorded as a root pathogen on Medicago in Australia. *Acrocalymma* species show cosmopolitan distribution worldwide (e.g., Australia, China, Egypt, India, Spain, Thailand, and the United States). Their host specificity is also not yet determined and has been recorded from various host families (e.g., Amaranthaceae, Arecaceae, Cucurbitaceae, Cycadaceae, Fagaceae, Lamiaceae, Magnoliaceae, and Moraceae) (Trakunyingcharoen et al. 2014; Jayasiri et al. 2019; Dong et al. 2020; Mortimer et al. 2021; Tennakoon et al. 2021; Calabon et al. 2023; Konta et al. 2023). Most *Acrocalymma* species have been recorded as coelomycetous, and two species have sexual morph, *A. pterocarpi* and *A. hongheense* (Jayasiri et al. 2019; Mortimer et al. 2021). The asexual morph exhibits papillate pycnidia and aseptate, hyaline conidia, which have appendages (Trakunyingcharoen et al. 2014; Jayasiri et al. 2019; Dong et al. 2020), whereas the sexual morph has immersed or semi-immersed, globose to subglobose, ostiolate ascomata, cylindric-clavate asci, and hyaline, fusiform, 1-septate ascospores with distinct sheath (Jayasiri et al. 2019; Mortimer et al. 2021). To date, there are 19 *Acrocalymma* species in Species Fungorum (2025).

### ﻿ Acrocalymma ampeli

Taxon classificationFungiPleosporalesDidymosphaeriaceae

Tennakoon, C.H. Kuo & K.D. Hyde, Fungal Diversity: 17 (2021)

6B05D079-473E-5F5C-A89F-2C325BB80EC1

Index Fungorum: IF555312

Facesoffungi Number: FoF09315

Fig. 6

#### Description.

***Saprobic*** on a dead leaf of *Livistona chinensis* (Arecaceae). **Sexual morph**: Undetermined. **Asexual morph**: ***Conidiomata*** 90–130 × 130–170 µm (*x̄* = 117 × 153 μm, *n* = 10), pycnidial, dark brown to black, solitary or clustered, immersed to semi-immersed, erumpent through host surface, unilocular, globose to subglobose, ostiolate. ***Conidiomatal wall*** 17–23 μm wide (*x̄* = 19 μm), composed of 4–5 layers of cells with *textura angularis*, cells towards the inside hyaline, and at the outside light brown. ***Conidiophores*** reduced to conidiogenous cells. ***Conidiogenous cells*** 5–8 × 4–7 µm (*x̄* = 6.7 × 5.8 μm, *n* = 20), hyaline, ampulliform to doliiform, phialidic, smooth-walled. ***Conidia*** 16–20 × 5–6 µm (*x̄* = 17.5 ×5.5 μm, *n* = 20), hyaline, cylindrical to fusoid, apex obtuse, protuberant and with a rounded hilum at base, aseptate, straight, thin-walled, with flaring mucoid apical appendage at lower end (3–4 µm diam.), visible in water mounts.

#### Material examined.

China • Yunnan Province, Kunming, on a dead leaf of *Livistona chinensis* (Arecaceae), 15 June 2017, D. S. Tennakoon, DST016 (SZU25-013, new host record) • *ibid.* 17 August 2017, DST011 (SZU25-014).

#### Known hosts.

*Livistona chinensis* and *Ficus ampelas* (Tennakoon et al. 2021; this study).

#### Known distribution.

China (Tennakoon et al. 2021; this study).

#### Notes.

*Acrocalymma ampeli* was introduced by Tennakoon et al. (2021) from dead leaves of *Ficus ampelas* (Moraceae). The morphology of our collection (SZU25-013 and SZU25-014) resembles *A. ampeli* by having pycnidial, dark brown to black, immersed to semi-immersed conidiomata, ampulliform to doliiform conidiogenous cells, and aseptate, cylindrical to fusoid-shaped conidia (Tennakoon et al. 2021). Multi-gene phylogeny also indicates that our collection clusters with *A. ampeli* isolates (MFLUCC 20-0159 and NCYUCC 19-0288) in a well-supported clade (100% ML, 1.00 BYPP). Therefore, based on morphological similarities and phylogeny support, we identified our collection as *A. ampeli* from a different host (*Livistona chinensis*) in China.

**Figure 6.**
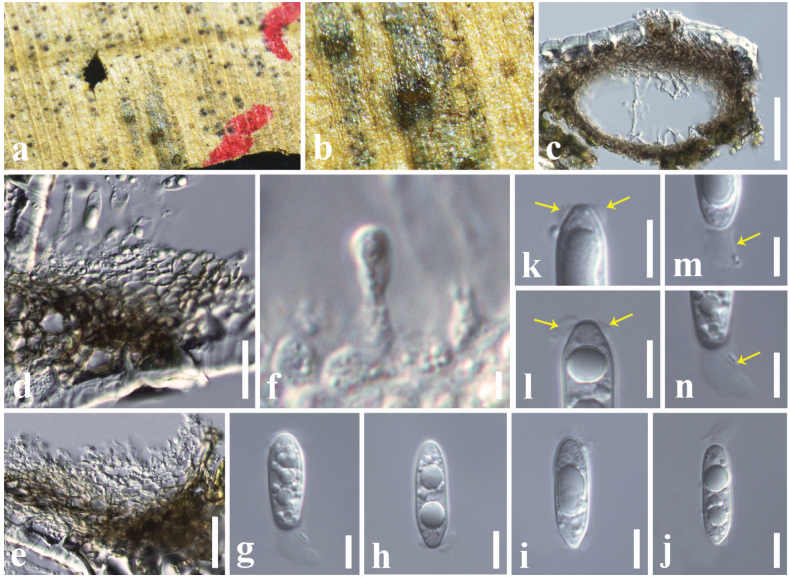
*Acrocalymma ampeli* (SZU25-013, new host record). a, b. Appearance of conidiomata on the host; c. Vertical section of a conidioma; d, e. Conidiomatal wall; f. Conidiogenous cells with bearing conidia; g–j. Conidia; k, l. Protruding hila at base (shows in yellow arrows); m, n. Mucoid apical appendages (shows in yellow arrows). Scale bars: 50 µm (c); 15 µm (d, e); 6 µm (f–j); 5 µm (k–n).

### ﻿ Acrocalymma poaceicola

Taxon classificationFungiPleosporalesDidymosphaeriaceae

Tennakoon & S. Hongsanan, sp. nov.

A17F7B5B-F98F-5EF7-A5AE-F4E288EA42B6

Index Fungorum: IF903948

Facesoffungi Number: FoF17789

Fig. 7

#### Etymology.

Named after the host family (Poaceae) where this fungus was collected.

#### Holotype.

SZU25-015.

#### Description.

***Saprobic*** on a dead leaf of *Arundo pliniana* (Poaceae). **Sexual morph**: Undetermined. **Asexual morph**: ***Conidiomata*** 60–70 × 80–110 µm (*x̄* = 64 × 97 μm, *n* = 10), pycnidial, dark brown to black, solitary or clustered, immersed to semi-immersed, erumpent through host surface, unilocular, globose to subglobose, ostiolate. ***Conidiomatal wall*** 15–20 μm wide (*x̄* = 17 μm), 3–4 layers of irregular cells arranged in a *textura angularis*, cells towards the inside hyaline, at the outside, light brown, thick-walled. ***Conidiophores*** reduced to conidiogenous cells. ***Conidiogenous cells*** 5–10 × 2.5–5 µm (*x̄* = 7.3 × 3.2 μm, *n* = 20), phialidic, hyaline, smooth, ampulliform to doliiform, proliferating with visible periclinal thickening at apex. ***Conidia*** 24–32 × 6–7.2 µm (*x̄* = 30 × 6.8 μm, *n* = 20), hyaline, cylindrical with an obtuse apex, protuberant and with a rounded hilum at base, straight, aseptate, guttulate, smooth-walled, bearing a mucilaginous appendage (2–2.5 µm diam.) at the apex.

#### Material examined.

China • Yunnan Province, Kunming, on a dead leaf of *Arundo pliniana* (Poaceae), 12 July 2017, D. S. Tennakoon, DST010 (SZU25-015, **holotype**) • *ibid.* 21 July 2017, DST015 (SZU25-016, **paratype**) • *ibid.* 15 August 2017, DST017 (SZU25-017, **paratype**).

#### Notes.

The morphology of our collection (SZU25-015, SZU25-016, and SZU25-017) tallies with *Acrocalymma* species in having pycnidial conidiomata, hyaline, ampulliform to doliiform conidiogenous cells, and aseptate, cylindrical to fusoid conidia (Zhang et al. 2012; Hyde et al. 2013; Mortimer et al. 2021; Tennakoon et al. 2021). According to the multi-gene phylogeny (LSU, SSU, and ITS) here, our collection clusters with *A. bilobatum* isolates (MFLUCC 20-0125 and K.L. Chen L119) with 87% ML and 0.95 BYPP statistical support. In addition, our collection isolates group together with 100% ML and 1.00 BYPP statistical support. Our collection can be distinguished from *A. bilobatum* in having smaller conidiomata (60–70 × 80–110 µm vs 170–275 × 135–205 µm) and larger conidia (24–32 × 6–7.2 µm vs 7–12 × 2.5–4 µm) with bearing a mucilaginous appendage (Calabon et al. 2023). A comparison of the 524 nucleotides across the ITS (+5.8S) gene region of our collection (SZU25-015) and *A. bilobatum* (MFLUCC 20-0125) shows 12 base pair differences (2.29%). Therefore, we recognize that these three isolates belong to one species, which we introduce as a new species herein.

**Figure 7.**
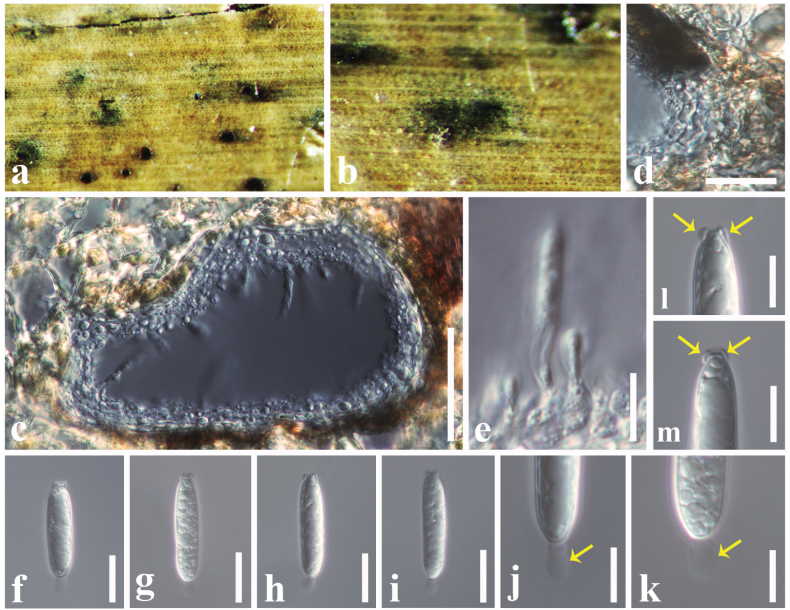
*Acrocalymma poaceicola* (SZU25-015, holotype). a, b. Appearance of conidiomata on the host; c. Vertical section of a conidioma; d. Conidiomatal wall; e. Conidiogenous cells with developing conidia; f–i. Conidia; j, k. Mucoid apical appendages (shows in yellow arrows); l, m. Protruding hila at base (shows in yellow arrows). Scale bars 30 µm (c); 15 µm (d); 15 µm (e–i); 10 µm (j–m).

### ﻿ Neokalmusia

Taxon classificationFungiPleosporalesDidymosphaeriaceae

H.A. Ariy. & K.D. Hyde, Fungal Diversity 68: 92 (2014)

59BF622A-2B55-599B-B71B-4099BB0D9288

#### Notes.

*Neokalmusia* was introduced by Ariyawansa et al. (2014b) to accommodate two species, *N. brevispora* and *N. scabrispora* (type species), which had been previously classified under *Kalmusia* (Tanaka et al. 2005; Zhang et al. 2009). *Neokalmusia* species have ascomata that are immersed to semi-immersed, globose to subglobose or oblong, with a clypeus-like structure; the asci are cylindric-clavate, with a long pedicel, and the ascospores are fusiform, yellowish-brown to reddish-brown and verrucose (Ariyawansa et al. 2014b; Thambugala et al. 2017; Hongsanan et al. 2020). Currently, ten *Neokalmusia* species are listed in Species Fungorum (2025). Interestingly, all *Neokalmusia* species have a host preference on Poaceae hosts (Ariyawansa et al. 2014b; Thambugala et al. 2017; Hongsanan et al. 2020; Hyde et al. 2020; Wanasinghe et al. 2022). In this study, we introduce a new host record of *N. arundinis* from another Poaceae host, *Sporobolus alterniflorus*.

### ﻿ Neokalmusia arundinis

Taxon classificationFungiPleosporalesDidymosphaeriaceae

Thambugala & K.D. Hyde, Mycosphere 8: 722 (2017)

F205877F-FE91-5A0C-BE85-33F794EF95ED

Index Fungorum: IF553161

Facesoffungi Number: FoF03219

Fig. 8

#### Description.

***Saprobic*** on a dead stem of *Sporobolus alterniflorus* (Poaceae). **Sexual morph**: ***Ascomata*** 300–370 × 200–250 μm (*x̄* = 340 × 230 μm, *n* = 6), immersed, appear as black dots, solitary, shiny, dark brown to black, sub-globose, uni-loculate, ostiolate. ***Peridium*** 14–22 μm wide (*x̄* = 18 μm), composed of 3–4 layers of brown to dark brown, cells of *textura angularis*, thin-walled. ***Hamathecium*** comprising 1.5–3 μm wide, numerous, cellular, pseudoparaphyses. ***Asci*** 75–95 × 7–9 μm (*x̄* = 85 × 8 μm, *n* = 20), 8-spored, bitunicate, fissitunicate, cylindric-clavate, long pedicellate, furcate at base, apically rounded with an indistinct ocular chamber. ***Ascospores*** 13–17 × 3.8–5.2 μm (*x̄* = 15 × 4.2 μm, *n* = 30), overlapping, 1–2-seriate, hyaline when immature, pale brown to dark brown at maturity, fusiform, 1-septate, distinctly constricted at the septum, straight or slightly curved, asymmetrical, upper cell shorter than lower cell, often enlarged near septum in the upper cell, smooth-walled, **Asexual morph**: Undetermined.

**Figure 8.**
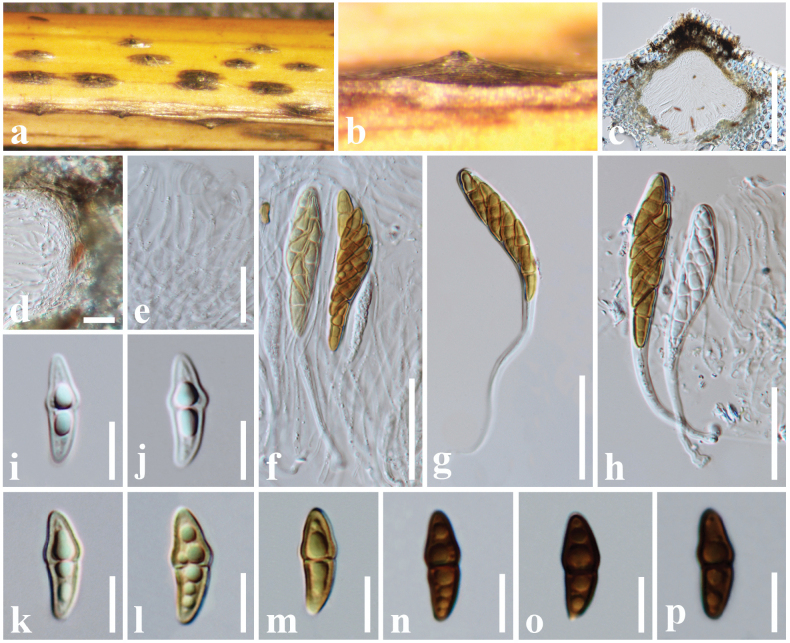
*Neokalmusia arundinis* (SZU25-019, new host record). a Appearance of ascomata on the host surface; b. Close up of an ascoma; c. Vertical section of an ascoma; d. Section through the peridium; e. Pseudoparaphyses; f–h. asci; i–p. Ascospores. Scale bars: 200 µm (c); 15 µm (d); 30 µm (e–h); 8 µm (i–p).

#### Material examined.

China • Yunnan Province, Kunming, on dead stem of *Sporobolus alterniflorus* (Poaceae), 28 December 2024, D.S. Tennakoon, DSZ21 (SZU25-019, new host record).

#### Known hosts.

*Arundo pliniana*, *Panicum virgatum*, *Sporobolus alterniflorus* (Thambugala et al. 2017; Hongsanan et al. 2020; this study).

#### Known distribution.

China and Italy (Thambugala et al. 2017; Hongsanan et al. 2020; this study).

#### Notes.

*Neokalmusia arundinis* was introduced by Thambugala et al. (2017) from a dead stem of *Arundo pliniana* in Italy. The morphology of our collection (SZU25-019) tallies well with the type of *N. arundinis* (MFLU 16–2577) by having immersed, globose to sub-globose, scattered, dark brown ascomata with a clypeus, cylindric-clavate, long pedicellate asci, and fusiform, pale brown to brown, 1-septate ascospores (Thambugala et al. 2017). In addition, the sizes of ascomata (275–350 × 225–275 μm vs 300–370 × 200–250 μm), asci (60–85 × 8.5–10.5 μm vs 75–95 × 7–9 μm), and ascospores (11.8–16.2 × 4–5.4 μm vs 13–17 × 3.8–5.2 μm) also overlap between our collection and the type of *N. arundinis* (MFLU 16–2577). According to the multi-gene phylogeny, our collection groups with other *N. arundinis* isolates in a well-supported clade (75% ML, 1.00 BYPP). Therefore, we introduce our collection as a new host record of *N. arundinis* from *Sporobolus alterniflorus* in China.

### ﻿ Neptunomyces

Taxon classificationFungiPleosporalesDidymosphaeriaceae

M. Gonçalves, T. Vicente & A. Alves, MycoKeys 60: 37 (2019)

DC1F0FE6-A819-58AB-96C3-6E6E51E26C20

#### Notes.

Gonçalves et al. (2019) introduced *Neptunomyces*, which includes *N. aureus*, isolated from *Ulva* sp. in Portugal. *Neptunomyces* species have aseptate, golden yellow, subcylindrical conidia with rounded apices. Currently, five *Neptunomyces* species are listed in Index Fungorum (2025): *N. aureus*, *N. jeanbriggsiae*, *N. juncicola*, *N. litoralis*, and *N. soli*. In this study, we introduce another *Neptunomyces* species, *N. chinensis*, from *Phragmites australis* in China.

### ﻿ Neptunomyces chinensis

Taxon classificationFungiPleosporalesDidymosphaeriaceae

Tennakoon & S. Hongsanan, sp. nov.

703D3E56-7EDC-5878-8D11-F6B1069850B3

Index Fungorum: IF903947

Facesoffungi Number: FoF17788

Fig. 9

#### Etymology.

Named after the country (China) where this fungus was collected.

#### Holotype.

SZU25-020.

#### Description.

***Saprobic*** on dead leaf of *Phragmites australis* (Poaceae). **Sexual morph**: Undetermined. **Asexual morph**: Coelomycetous. ***Conidiomata*** 90–110 × 80–150 µm (*x̄* = 102 × 120 µm, *n* = 10), pycnidial, immersed to semi-immersed, dark brown to black, solitary to aggregated, globose to sub-globose, visible as dots on host surface. ***Conidiomatal wall*** 15–20 μm wide (*x̄* = 16 µm), thick-walled, composed of several layers of brown to dark brown pseudoparenchymatous cells, fusing at the outside indistinguishable from the host tissues. ***Conidiophores*** reduced to conidiogenous cells. ***Conidiogenous cells*** 4–7 × 3–4 µm (*x̄* = 4.8 × 3.5 µm, *n* = 20), discrete, hyaline, globose to doliiform, holoblastic. ***Conidia*** 6.5–8 × 4–5 (*x̄* = 6.8 × 4.5 µm, *n* = 40) µm, hyaline to pale brown, ellipsoidal to limoniform, apex acute to apiculate, widest in the middle, tapering towards a narrowly truncate base, guttulate, smooth-walled.

**Figure 9.**
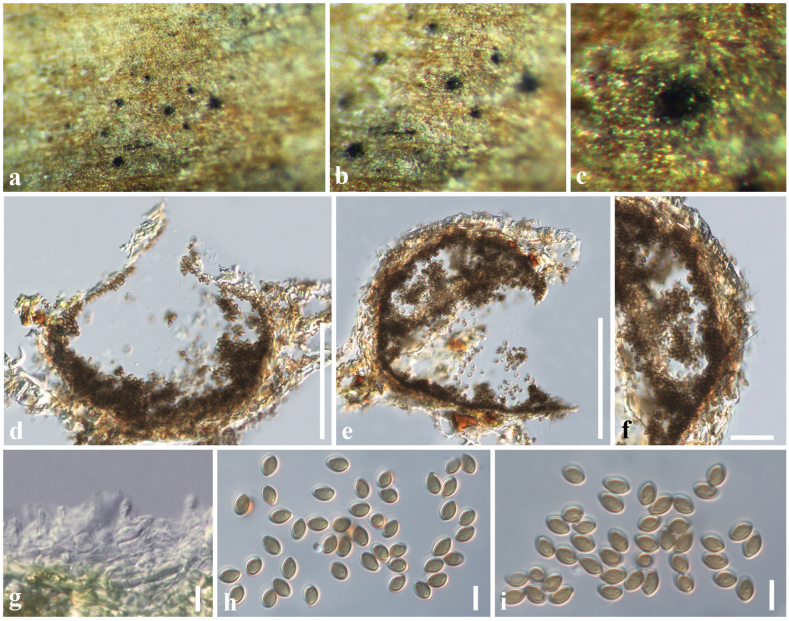
*Neptunomyces chinensis* (SZU25-020, holotype). a, b. Appearance of conidiomata on host; c. Close-up of conidioma; d, e. Vertical sections of conidiomata; f. Conidiomatal wall; g. Conidiogenous cells with developing conidia; h, i. Conidia. Scale bars: 60 µm (d, e); 15 µm (f); 5 µm (g–i).

#### Material examined.

China • Guangdong Province, Shenzhen, on a dead leaf of *Phragmites australis* (Poaceae), 20 December 2024, D.S. Tennakoon, SZD 12 (SZU25-020, **holotype**), ex-type living culture, MBSZU 25-028 • *ibid.* 23 December 2024, SZD15 (SZU25-021, **paratype**), living culture, MBSZU 25-029.

#### Notes.

According to the multi-gene phylogeny, our collection (SZU25-020 and SZU25-021) constitutes an independent lineage sister to *Neptunomyces juncicola* (CPC 45436) with robust statistical support (97% ML, 1.00 BYPP). In addition, new isolates cluster together with strong support (100% ML, 1.00 BYPP). Our collection can be distinguished from *N. juncicola* by conidial characteristics; *N. juncicola* has subcylindrical to fusoid-ellipsoid conidia with sub-obtuse apex, whereas our collection has ellipsoidal to limoniform conidia with acute to apiculate apex (Crous et al. 2024). Our collection differs from *N. juncicola* by having smaller conidiomata (90–110 × 80–150 µm vs 180–220 µm). However, SZU25-020 and SZU25-021 have some morphological similarities with *N. soli* (immersed to semi-immersed, globose to sub-globose conidiomata and doliiform conidiogenous cells and hyaline to pale brown conidia), but differ by conidia sizes (6.5–8 × 4–5 µm vs 5–6.5 × 3–5 µm) (Yasanthika et al. 2024). In addition, a comparison of the 587 nucleotides across the ITS (+5.8S) gene region of our collection (SZU25-020) and *N. soli* (MFLUCC 24-0272) shows 19 base pair differences (3.23%). Therefore, based on both morphological and phylogenetic evidence, we introduce our collection as a new species, *N. chinensis*, from China.

### ﻿ Paraconiothyrium

Taxon classificationFungiPleosporalesDidymosphaeriaceae

Verkley, Stud. Mycol. 50(2): 327 (2004)

32A6DD7D-7DC8-5B67-845D-028CB14D7976

#### Notes.

Verkley et al. (2004) introduced *Paraconiothyrium*, which includes four species: *P. estuarinum* (type species), *P. brasiliense*, *P. cyclothyrioides*, and *P. fungicola*. *Paraconiothyrium* species have variable morphological characteristics, including eustromaticto pycnidial conidiomata, phialidic or annelidic conidiogenous cells, smooth-walled or minutely warted, hyaline or brown conidia (Verkley et al. 2004; Damm et al. 2008; Paul and Lee 2014). The life modes of *Paraconiothyrium* can vary, as they are primarily reported as saprobes, with some species also being identified as endophytes and pathogens (Khan et al. 2012; Nakashima et al. 2019; Wang et al. 2021; Guarnaccia et al. 2022; Tennakoon et al. 2022). They also have diverse bioactive functions with potential applications in agriculture, medicine, and industrial sectors (Wang et al. 2021). To date, 28 species of *Paraconiothyrium* have been listed in Species Fungorum (2025). In this study, we introduce a new host record of *P. archidendri* from *Citrus maxima* in China.

### ﻿ Paraconiothyrium archidendri

Taxon classificationFungiPleosporalesDidymosphaeriaceae

Verkley, Göker and Stielow, Persoonia 32: 37 (2014)

B8784CB5-B500-5612-8574-EDF8D87C32B4

Index Fungorum: IF800761

Facesoffungi Number: FoF05243

Fig. 10

#### Description.

***Saprobic*** on a dead stem of *Citrus maxima* (Rutaceae). **Sexual morph**: Undetermined. **Asexual morph**: Coelomycetous. ***Conidiomata*** 100–150 × 100–170 µm (*x̄* = 105 × 135 µm, *n* = 10), pycnidial, immersed to semi-immersed, dark brown to black, solitary to aggregated, globose to sub-globose. ***Conidiomatal wall*** 15–30 μm wide (*x̄* = 17 µm), thick-walled, composed of several layers of brown to dark brown pseudoparenchymatous cells, cells towards the inside hyaline, arranged in a *textura angularis*, fusing at the outside indistinguishable from the host tissues. ***Conidiophores*** reduced to conidiogenous cells. ***Conidiogenous cells*** 6–8 × 3–4 µm (*x̄* = 6.4 × 3.2 µm, *n* = 20), discrete, hyaline, globose to doliiform, holoblastic. ***Conidia*** 5.8–7 × 3–4 (*x̄* = 6.3 × 3.2 µm, *n* = 40) µm, variable in shape, subglobose to ellipsoid, rarely obovoid, ends rounded, aseptate, initially hyaline, becoming olivaceous-brown at maturity, contents with several small oil droplets, smooth-walled.

#### Known hosts.

*Acer pentaphyllum*, *Alliaria petiolate*, *Cinnamomum camphora*, *Citrus maxima*, *Coffea arabica*, *Ginkgo biloba*, *Magnolia* sp., *Picea glauca*, *Pinus tabulaeformis*, *Pithecolobium bigeminum* and *Prunus salicina* (Verkley et al. 2004, 2014; Damm et al. 2008; Paul and Lee 2014; Nakashima et al. 2019; Tennakoon et al. 2022; this study).

#### Known distribution.

Brazil, Canada, China, Japan, Korea, Myanmar, South Africa, and the United States (Verkley et al. 2004, 2014; Damm et al. 2008; Paul and Lee 2014; Nakashima et al. 2019; Tennakoon et al. 2022; this study).

#### Material examined.

China • Guangdong Province, Shenzhen, on dead stem of *Citrus maxima* (Rutaceae), 21 December 2024, D. S. Tennakoon, DC030 (SZU25-018, new host record); living culture MBSZU 25-027.

#### Notes.

*Paraconiothyrium archidendri* was initially introduced by Verkley et al. (2014) from *Pithecellobium bigeminum* in Myanmar. A multi-gene phylogeny indicates that our collection (SZU25-018) groups with other *P. archidendri* isolates, particularly closely related to the isolate CBS 168.77. Our collection shares similar morphological characteristics with *P. archidendri* by having pycnidial, immersed to semi-immersed, dark brown to black conidiomata, globose to doliiform conidiogenous cells, and subglobose to ellipsoid, olivaceous-brown, aseptate conidia (Damm et al. 2008; Paul and Lee 2014; Verkley et al. 2014; Nakashima et al. 2019; Tennakoon et al. 2022). Thus, we identify our collection as *Paraconiothyrium archidendri* with a new host occurrence from *Citrus maxima* in China.

**Figure 10.**
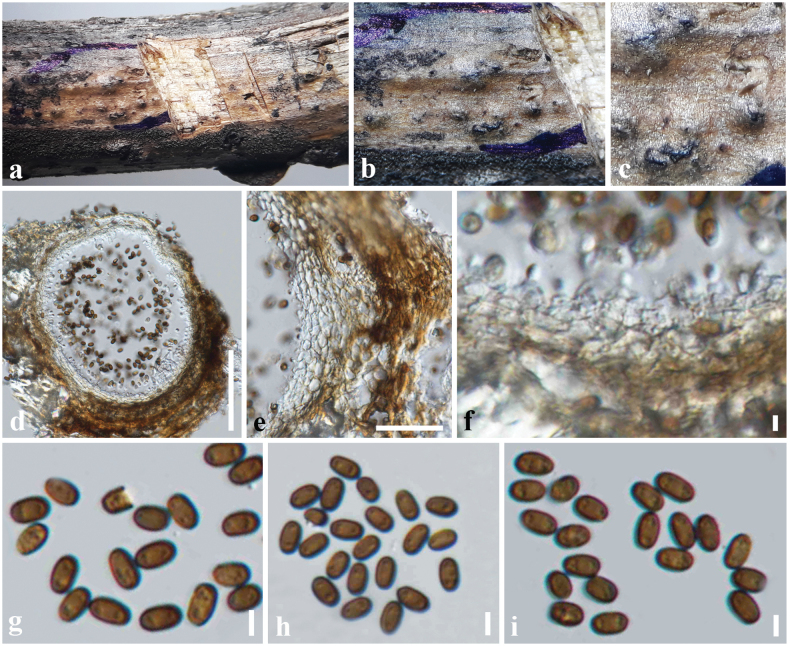
*Paraconiothyrium archidendri* (SZU25-018, new host record). a. Conidiomata on a dead stem of *Citrus maxima*; b, c. Close-up of conidiomata on the host; d. Section through conidioma; e. Conidiomatal wall; f. Conidiogenous cells with developing conidia; g–i. Conidia. Scale bars: 50 µm (d); 20µm (e); 5 µm (f–i).

### ﻿ Pseudopithomyces

Taxon classificationFungiPleosporalesDidymosphaeriaceae

Ariyaw. & K.D. Hyde, Fungal Diversity 75: 64 (2015)

FC4788BC-9BA1-58FE-8E6E-A818F91EBF76

#### Notes.

Ariyawansa et al. (2015) introduced this genus to place *Pseudopithomyces chartarum* as the type species. *Pseudopithomyces* has a cosmopolitan distribution worldwide (Hyde et al. 2017; Wu et al. 2023; Farr and Rossman 2025). Species have fusiform, verruculose, dark conidia and produce brown to black colonies on the host (Ariyawansa et al. 2015; Jayasiri et al. 2019; Tennakoon et al. 2021). Currently, 13 *Pseudopithomyces* species are listed in Species Fungorum (2025). In this study, we introduce a new host record of *P. chartarum* from a dead leaf of *Hedychium coronarium* (Zingiberaceae).

### ﻿ Pseudopithomyces chartarum

Taxon classificationFungiPleosporalesDidymosphaeriaceae

(Berk. & M.A. Curtis) Jun F. Li, Ariyaw. & K.D. Hyde, Fungal Divers 75: 66 (2015)

EB5CF47E-ABC8-50AB-BE7B-FA897BDB0980

Index Fungorum: IF551393

Facesoffungi Number: FoF00938

Fig. 11

#### Description.

***Saprobic*** on a dead leaf of *Hedychium coronarium* (Zingiberaceae). **Sexual morph**: Undetermined. **Asexual morph**: Hyphomycetous. Colonies effuse, scattered, powdery, dark brown to black. ***Vegetative hyphae*** superficial or partly immersed in the substrate, composed of septate, branched, smooth, thin-walled hyphae. ***Conidiophores*** mononematous, micronematous, mostly intercalary, sometimes denticulate, aseptate. ***Conidiogenous cells*** with 2 µm broad conidial attachment, terminal, hyaline, globose or subglobose, integrated, hyaline to pale brown. ***Conidia*** 15–21 × 8–10 μm (*x̄* = 17 × 9.1 μm, *n* = 40), solitary, initially light brown, becoming brown to dark brown at maturity, obovate to oblong, verruculose to spinulose, muriform, 3–4 vertical septa, mostly 1–2 longitudinal dark septa, darken and slightly constricted at the septa, thick-walled.

#### Material examined.

China • Guangdong Province, Shenzhen, on dead leaf of *Hedychium coronarium* (Zingiberaceae), 15 December 2024, D.S. Tennakoon, DSZ10 (SZU25-022, new host record), living culture, MBSZU 25-030.

#### Known hosts.

*Bauhinia* spp., *Centrosema pubescens*, *Ceratonia siliqua*, *Chloris gayana*, *Chrysanthemum coronarium*, *Cirsium arvense*, *Cocos* spp., *Colocasia* spp., *Commelina benghalensis*, *Conyza bonariensis*, *Crotalaria pseudospartium*, *Crotalaria striata*, *Cunninghamia lanceolata*, *Cupania macrophylla*, *Cynodon* spp., *Dactylis glomerata*, *Daucus* spp., *Descurainia sophia*, *Desmodium* spp., *Digitaria* spp., *Dolichos* spp., *Eichhornia crassipes*, *Elegia* spp., *Eucalyptus* spp., *Foeniculum vulgare*, *Glycine* spp., *Gossypium* spp., *Guazuma ulmifolia*, *Harpullia* spp., *Ipomoea* spp., *Juncus roemerianus*, *Leucaena* spp., *Macaranga tanarius*, *Magnolia grandiflora*, *Malus* spp., *Medicago* spp., *Miscanthus* spp., *Morus* spp., *Musa* spp., *Oryza sativa*, *Pandanus tectorius*, *Panicum* spp., *Phyllostachys* spp., *Radermachera sinica*, and *Zea mays* (Hyde et al. 2017; Jayasiri et al. 2019; Tennakoon et al. 2021; Farr and Rossman 2025; this study).

#### Known distribution.

Australia, Austria, Brazil, Canada, China, Cuba, Japan, Ghana, Greece, India, Indonesia, Kenya, Malaysia, Myanmar, Nicaragua, New Zealand, Papua New Guinea, Poland, South Africa, Sudan, Thailand, Venezuela, the United States (Hyde et al. 2017; Jayasiri et al. 2019; Tennakoon et al. 2021; Farr and Rossman 2025; this study).

**Figure 11.**
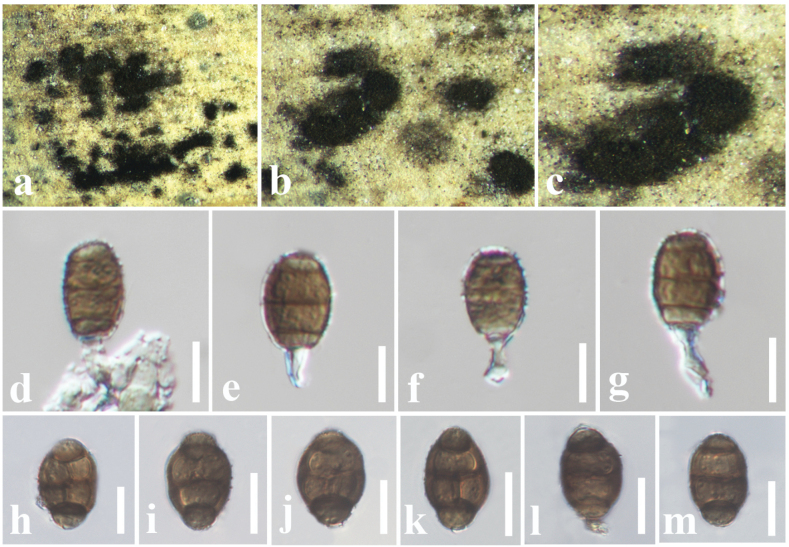
*Pseudopithomyces chartarum* (SZU25-022, new host record). a–c. Appearance of colonies on host surface; d–g. Conidiogenous cells bearing conidia; h–m. Conidia. Scale bars: 10 µm (d–m).

#### Notes.

Due to the morphological characteristics largely overlapping with those of *Pseudopithomyces chartarum* isolates, we report our collection (SZU25-022) as a new host record of *P. chartarum* from dead leaves of *Hedychium coronarium* (Zingiberaceae). For instance, our collection resembles *P. chartarum*, having powdery, dark brown to black colonies on the host surface and brown, obovate to oblong, muriform conidia (Ariyawansa et al. 2015; Jayasiri et al. 2019; Samaradiwakara et al. 2022; Wu et al. 2023). Phylogeny also shows that our collection groups with other *P. chartarum* isolates in a well-supported clade (90% ML, 0.98 BYPP).

### ﻿ Spegazzinia

Taxon classificationFungiPleosporalesDidymosphaeriaceae

Sacc. Padova: [1] (1879)

CE013A4B-2B02-5C12-80F4-7BEB26386947

#### Notes.

*Spegazzinia* is a diverse, hyphomycetous genus introduced by Saccardo (1880) to include *S. ornata* as the type species. *Spegazzinia* species have been reported mainly as saprobes on litter of tropical, subtropical, and temperate vascular plants (Matsushima 1980; Lu et al. 2000; Manoharachary and Kunwar 2010; Thambugala et al. 2017). Additionally, some have been identified as endophytes (e.g., *S. tessarthra* and *S. bromeliacearum*) (Manish et al. 2014; Crous et al. 2019). Apart from that, *Spegazzinia* species have also been found in soil (Ellis 1971). The morphological characteristics of *Spegazzinia* are quite distinct from those of other hyphomycetous genera due to its pleomorphism (Mena-Portales et al. 2017). They have two types of conidia in the same mycelium, such as *α* and *β* conidia. Some species have spines in both types of conidia, while some taxa do not bear spines. Currently, 28 *Spegazzinia* species are listed in Species Fungorum (2025). In this study, we introduce a new host record of *S. deightonii* from a dead leaf of *Arundo pliniana* in China.

### ﻿ Spegazzinia deightonii

Taxon classificationFungiPleosporalesDidymosphaeriaceae

(S. Hughes) Subram., J. Indian Bot. Soc. 35: 78 (1956)

561F35A5-30F1-5EE1-9A8C-F171241166FE

Index Fungorum: IF306062

Facesoffungi Number: FoF07238

Fig. 12

#### Description.

***Saprobic*** on a dead leaf of *Arundo pliniana* (Poaceae). **Sexual morph**: Undetermined. **Asexual morph**: Hyphomycetous. ***Sporodochia*** 1–2 mm diam., dark, black, dense, powdery, velvety. ***Conidiophores*** give rise to two types of conidia referred to here as *α* and *β*. *Conidiophores* of *α* conidia up to 75–90 × 1–2 μm (*x̄* = 80 × 1.6 μm, *n* = 20) long, erect or flexuous, narrow, verruculose, unbranched, base light brown, upper part dark brown. *Conidiogenous cell* development basauxic, forming a single, terminal holoblastic conidium at the apex of the conidiophore. Conidial development holoblastic. ***Conidia*** two types: ***α conidia*** 15–27 × 16–24 μm (*x̄* = 25 × 21 μm, *n* = 25), stellate, solitary, globose to variously shaped, with spines 4–6 μm long, 4–8-celled, deeply constricted at the septa. ***β conidia*** 16–22 × 10–15 μm (*x̄* = 19 × 14 μm, *n* = 25), disc-shaped, initially hyaline, light brown to dark brown at maturity, 8-celled, flat from both sides, frequently with attached conidiogenous cells when splitting from the conidiophores.

#### Known hosts.

*Arundo pliniana*, *Areca catechu*, *Cocos nucifera*, *Hedychium coronarium*, *Musa* sp., and *Panicum maximum* (Matsushima 1980; Lu et al. 2000; Tianyu 2009; Samarakoon et al. 2020; Tennakoon et al. 2022; Farr and Rossman 2025; this study).

#### Known distribution.

China and Thailand (Matsushima 1980; Lu et al. 2000; Tianyu 2009; Samarakoon et al. 2020; Tennakoon et al. 2022; Farr and Rossman 2025; this study).

#### Material examined.

China • Guangdong Province, Shenzhen, on dead leaf of *Arundo pliniana* (Poaceae), 21 December 2024, D. S. Tennakoon, DROD009 (SZU25-023, new host record); living culture, MBSZU 25-031.

#### Notes.

The morphological characteristics of our collection (SZU25-023) resemble those of *Spegazzinia deightonii*, as it has 8-celled, disk-shaped, dark brown, spiny conidia (Tanaka et al. 2015; Samarakoon et al. 2020; Tennakoon et al. 2022). Phylogeny also indicates that our collection clusters with other *S. deightonii* isolates in a well-supported clade (92% ML, 0.95 BYPP), and makes a close phylogenetic relationship with the isolate MFLUCC 20-0002 (96% ML, 0.96 BYPP). Therefore, we identify our collection as an isolate of *S. deightonii* from a dead leaf of *Arundo pliniana* in China.

**Figure 12.**
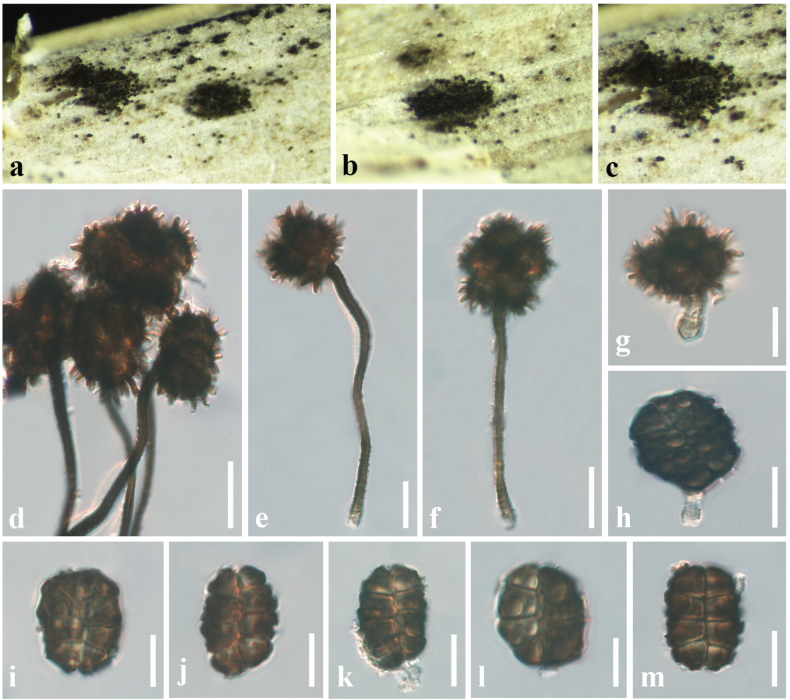
*Spegazzinia deightonii* (SZU25-023, new host record). a. Sporodochia on a dead leaf of *Arundo pliniana*; b, c. Close-up of sporodochia; d–f. *α* Conidia with conidiophores; g, h. *α* Conidia with conidiogenous cells; i–m. *β* Conidia. Scale bars: 15 µm (d–h); 10 µm (i–m).

## ﻿Discussion

Fungal taxonomy has undergone significant evolution over the last two decades, transitioning from traditional morphology-based classification to the current era of DNA-based fungal classification (Tedersoo et al. 2020; Lücking et al. 2021). Initially, morphological characteristics served as the primary criteria for fungal classification, which were mostly represented by line drawings, taxonomic keys, descriptions, and illustrations (Ellis 1971; Nagraj 1978; Sutton 1980). The traditional approach persisted for centuries and was largely limited by factors such as phenotypic plasticity, which has led to misinterpretations and taxonomic confusions (Alster et al. 2021). Consequently, morphological characteristics combined with a DNA-based taxonomic approach have emerged as the main research tool for fungal classification and have facilitated the resolution of species boundaries with numerous novel fungal discoveries (White et al. 1990; Tanaka et al. 2015; Hyde et al. 2020; Crous et al. 2021; Mortimer et al. 2021; Tennakoon et al. 2024). In particular, molecular techniques have facilitated the construction of fungal phylogenies, the identification of evolutionary patterns, and the discovery of cryptic species (Hyde et al. 2017; Fontecha et al. 2019; Hongsanan et al. 2020). However, the accurate identification of fungal species remains a significant challenge. To overcome these challenges, mycologists have made significant strides in resolving fungal taxonomy across various taxonomic levels, including orders, families, genera, and species. For instance, mycologists have identified the class Dothideomycetes, comprising 46 orders, 231 families, 2,346 genera, and 32,621 species (Hongsanan et al. 2020; Wijayawardene et al. 2022; Bánki et al. 2023).

We describe and illustrate two new fungal species (*Acrocalymma poaceicola* and *Neptunomyces chinensis*) and five new host records (*A. ampeli*, *Neokalmusia arundinis*, *Paraconiothyrium archidendri*, *Pseudopithomyces chartarum*, and *Spegazzinia deightonii*) in Acrocalymmaceae and Didymosphaeriaceae families (Pleosporales, Dothideomycetes) based on morphological and molecular analyses (Figs 6–12). All species were collected from forest plant litter. The newly proposed species, *Acrocalymma poaceicola* and *Neokalmusia chinensis*, are distinguished from related taxa through both distinct morphological characteristics and molecular phylogenetic evidence. Additionally, the morphological features of the new host records align with those of their respective type specimens, exhibiting dimensional overlap.

The species of Didymosphaeriaceae have a cosmopolitan distribution worldwide and have been recorded from a vast range of host families (Ariyawansa et al. 2014a, b; Hyde et al. 2013; Liu et al. 2015; Wanasinghe et al. 2016; Phookamsak et al. 2019; Tennakoon et al. 2022; Farr and Rossman 2025). Of them, some Didymosphaeriaceae genera are vastly diverse with many species (e.g, *Didymosphaeria*: 195 species, *Paraphaeosphaeria*: 40 species, *Montagnula*: 49 species, *Kalmusia*: 31 species, *Paraconiothyrium*: 28 species, *Karstenula*: 23 species, *Phaeodothis*: 22 species, *Spegazzinia*: 28 species, *Pseudocamarosporium*: 15 species, *Pseudopithomyces*: 15 species, *Letendraea*: 12 species, *Neokalmusia*: 10 species, *Tremateia*: 10 species, *Paracamarosporium*: 9 species, *Chromolaenicola*: 8 species, *Laburnicola*: 8 species, *Deniquelata*: 7 species, *Julella*: 7 species, *Curreya*: 6 species, *Neptunomyces*: 5 species). Some genera consist of less than 5 species (e.g., *Alloconiothyrium*: 3 species, *Austropleospora*: 3 species, *Verrucoconiothyrium*: 3 species, *Bimuria*: 2 species, *Cylindroaseptospora*: 2 species, *Didymocrea*: 2 species, *Paramassariosphaeria*: 2 species). In addition, some genera remain monotypic, thus additional collections are needed to facilitate their expansion (e.g., *Barria*, *Kalmusibambusa*, *Lineostroma*, *Vicosamyces*, *Xenocamarosporium*). Despite having a large number of genera in Didymosphaeriaceae, some are still lacking molecular data (e.g., *Barria*, *Lineostroma*). Thus, future collections are essentially required to resolve their phylogenetic relationships in Didymosphaeriaceae.

By contrast, Acrocalymmaceae is monotypic, consisting only of *Acrocalymma*, which has 19 species. Most of the Acrocalymmaceae taxa play a crucial role in various ecological niches (e.g., terrestrial, freshwater, soil) as saprobes (Trakunyingcharoen et al. 2014; Jayasiri et al. 2019; Mortimer et al. 2021; Tennakoon et al. 2021; Konta et al. 2023). In addition, they are known to act as pathogens as well (e.g., *A. medicaginis*, *A. vagum*) (Trakunyingcharoen et al. 2014). The host specificity of Acrocalymmaceae still needs to be investigated, as they have been reported from a wide host range (e.g., *Anomianthus dulcis*, *Arenga pinnata*, *Cycas calcicole*, *Ficus ampelas*, *Magnolia* spp., and *Quercus glauca*) (Trakunyingcharoen et al. 2014; Jayasiri et al. 2019; Das et al. 2020; Mortimer et al. 2021; Tennakoon et al. 2021; Calabon et al. 2023; Konta et al. 2023). Nonetheless, it is worthwhile to collect additional fungal samples from various habitats and ecological niches to facilitate the continued expansion of this genus.

## Supplementary Material

XML Treatment for Acrocalymma

XML Treatment for Acrocalymma ampeli

XML Treatment for Acrocalymma poaceicola

XML Treatment for Neokalmusia

XML Treatment for Neokalmusia arundinis

XML Treatment for Neptunomyces

XML Treatment for Neptunomyces chinensis

XML Treatment for Paraconiothyrium

XML Treatment for Paraconiothyrium archidendri

XML Treatment for Pseudopithomyces

XML Treatment for Pseudopithomyces chartarum

XML Treatment for Spegazzinia

XML Treatment for Spegazzinia deightonii

## ﻿Additional information

### Use of AI

No use of AI was reported.

### Author ORCIDs

Danushka S. Tennakoon https://orcid.org/0000-0003-2306-1255

Nimali I. de Silva https://orcid.org/0000-0002-1577-280X

Sinang Hongsanan https://orcid.org/0000-0003-0550-3152

Ning Xie https://orcid.org/0000-0002-5866-8535

### Data availability

All of the data that support the findings of this study are available in the main text.
